# Posters

**DOI:** 10.1038/sj.bjc.6601928

**Published:** 2004-06-22


### P151

### VALIDATION OF A 96-WELL PLATE BASED ATP ASSAY FOR DETERMINING CHEMOSENSITIVITY OF LEUKAEMIC BLAST CELLS

TB Davis [1], CJ Williamson [3], JM Sargent [3], F van Delft [2], V Saha [2], SP Joel [1]
[1] London, United Kingdom, United Kingdom, [2] London, United Kingdom, United Kingdom

The MTT assay has been used for the *in vitro* determination of chemosensitivity in childhood ALL blast cells, but can be limited by the number of available cells. We have investigated the ViaLight™ HS ATP assay (Cambrex Bioscience, Wokingham, UK), a more sensitive technique that uses up to 10 times fewer blast cells than the traditional MTT assay, for this purpose. Ficoll density separated blast cells from bone marrow or peripheral blood from children diagnosed with ALL or AML were thawed, washed with RPMI 1640 with 20% FBS and resuspended in RPMI 1640 with 10% FBS, 100IU penicillin and 100µg/ml streptomycin. Cell count and viability were measured using the trypan blue exclusion method and $5x10^5$ cells/ml plated into 96-well plates. Cells were then exposed to cytotoxic drugs at a minimum of 4 concentrations in triplicate for 48 or 96 hours (depending on the drug) in a humidified atmosphere of 5% $CO_2$ at $37^0$C. After drug exposure the medium was removed, cells were resuspended in 100 µl of RPMI 1640 with 10% FBS, and ATP released using 100µl nucleotide releasing reagent. After incubation at room temperature for 5 minutes plates were carefully mixed and 180µl transferred to opaque white walled microtitre plates. ATP concentration was determined immediately using a bioluminescence method. Results were expressed as % luminescence relative to control (untreated wells) and the $EC_{50}$ concentration calculated using Graphpad Prism software. The method was reproducible (e.g. $EC_{50}$ for etoposide and dexamethasone in a sample analysed 5 times $1.26 \pm 0.38$ and $0.018 \pm 0.004$ µg/mL respectively). Additionally, comparable results were obtained for samples analysed from frozen compared to the same sample analysed as fresh blasts in a different laboratory[2] (e.g. $EC_{50}$ for etoposide in 3 samples 1.26 vs. 1.30, 0.95 vs. 0.98 and 1.65 vs. 1.21 µg/mL). The method is now being used to determine chemosensitivity to a panel of established and novel agents in a paediatric leukaemia study.

### P152

### FIBULIN-1 AND -4 MRNA EXPRESSION LEVELS IN HUMAN COLORECTAL CANCER

IM Nolan , EJ Fox , C Moss , DT Leahy , R Geraghty , HE Mulcahy , JP Hyland , D Fennelly , K Sheahan , D O'Donoghue , WS Argraves , WM Gallagher
[1] United Kingdom, Department of Pharmacology, Department of Pathology and Core Technology Group, Conway Institute of Biomolecular and Biomediclal Research, University College Dublin, Dublin, [2] Ireland, Centre for Colorectal Disease, St. Vincent's University Hospital, Dublin, [3] Ireland, Department of Cell Biology and Anatomy, Medical University of South Carolina, South Carolina

The sequential progression of colorectal cancer offers unique possibilities for studying the genetic changes involved in tumour progression. In this study, we investigated the expression of two members of the fibulin family (fibulin-1 and -4) in colon cancer. Different fibulin variants demonstrate different roles in cancer and can act as either oncogenes or tumour suppressor genes. The aim of this work was to assess fibulin-1 and -4 mRNA expression, using TaqMan Real-time PCR assays (ABI Prism 7700 SDS), in a large panel of colon tumour versus matched normal tissues with a view to determining associations with tumour development and progression along with clinicopathological variables. In addition we have assessed fibulin-1 and -4 expression patterns in a large panel of tumour- and normal- derived cell lines from different tissues origins.

Our biopsy study revealed an increase in fibulin-4 expression (P=0.003, n=40 patients, Matched Wilcoxan test) in tumour samples compared to the corresponding matched normal tissue. When correlated with patient profiles, the comparisons that approached clinical significance were observed between increased fibulin-4 expression and lower polyp number (P=0.086, Wilcoxan test), as well as lymphovascular invasion (P=0.08, Wilcoxan test). We also noted a slight downregulation of fibulin-1 mRNA expression in tumour biopsies (n=38 patients, P=0.095, Matched Wilcoxan test).

These results confirm an involvement of fibulin family members in colon cancer. The expression of fibulin-1 is probably dependent on which isoforms are expressed since we have designed real-time PCR assays to detect fibulin-1C and –1D isoforms specifically and are in the process of reassessing the expression patterns of these splice variants in the same colon tissue pairs. We have also demonstrated elevated fibulin-4 expression in tumour biopsies, which is in accordance with the candidate oncogenic role previously suggested for this gene.

### P153

### IDENTIFICATION AND CHARACTERISATION OF PROTEINS INTERACTING WITH HUMAN DNA TOPOISOMERASE I

A McNamara , AJM Watson , JR Jenkins
United Kingdom, University of Liverpool, Liverpool

**Background:** Human topoisomerase I (Top I) is of considerable biomedical importance because it is the target of the camptothecin (CPT) family of anticancer drugs. Derivatives of CPT, irinotecan and topotecan are routinely used in clinic, primarily in the treatment of metastatic colorectal cancer. The aim of this study is to identify novel proteins interacting with Top I, which may reveal new potential chemotherapeutic targets.

**Methods:** Proteins that interact with Top I were identified using extracts from the human colon adenoma HCT116 cell line by immunoprecipitation, followed by mass spec (MALDI-TOF) analysis. Interactions were confirmed by counter immunoprecipitation.

**Results:** To date eighteen proteins have been identified as physically interacting with Top I. One of these is Hsp70, which is known to be associated with Top I. Seven of these interactions have been confirmed, which include the molecular chaperone Hsp90. This is the target of new up and coming anti cancer therapies. Using the Hsp90 inhibitor geldanamycin (GA) on the HCT116 cell line, we have disrupted the Top I and Hsp90 interaction. Upon addition of a Top I poison we see a synergistic effect on both cell killing and proliferation inhibition. Surprisingly we have also demonstrated that up to 48 hours after GA treatment Top I is not degraded. This is despite the fact that GA binding is thought to promote assembly of a super chaperone machine that favours client protein degradation (Scheider et al 1996).

**Conclusion**: We have identified a direct interaction between Top I and Hsp90, and found the combination of GA and Top I poisons act synergistically to both inhibit the growth of and kill colorectal cancer cells.

**Continuing Work:** Elucidation of the mechanism behind the synergistic effect observed when Top I and Hsp90 inhibitors are used in combination.

### P154

### QUANTITATIVE TRAIT LOCUS ANALYSIS REVEALS TWO INTRAGENIC SITES THAT INFLUENCE *O*6-ALKYLGUANINE-DNA ALKYLTRANSFERASE ACTIVITY IN PERIPHERAL BLOOD MONONUCLEAR CELLS

GP Margison , J Heighway , SJ Pearson , AC Povey , MF Santibáñez Koref6
United Kingdom, Institute of Human Genetics, University of Newcastle, Newcastle

The repair of specific types of DNA alkylation damage by $O^6$-alkylguanine-DNA alkyltransferase (MGMT) is a major mechanism of resistance to the carcinogenic and chemotherapeutic effects of certain alkylating agents. The levels of expression of MGMT normal and tumour tissues are thus of interest in relation to the prevention and treatment of cancer. MGMT expression in a given tissue varies widely between individuals but the underlying causes of this variability are not known. Here we investigate the contribution of variation at the DNA level on intra-individual differences in MGMT activity in peripheral blood mononuclear cells (PBMC). First we use an expressed single nucleotide polymorphism (SNP) to demonstrate that the two MGMT alleles are frequently expressed at different levels in PBMC, suggesting that there is a genetic component of inter-individual variation of MGMT levels that maps close to or within the MGMT locus. Next, we show by quantitative trait locus analysis using intragenic SNPs that there are at least two sites influencing interindividual variation in MGMT activity in PBMC. One of these sites is characterized by an SNP at the 3' end of the first intron and the second by two SNPs in the last exon. The latter two are in perfect disequilibrium and result both in amino acid substitutions; one of them, Ile143Val, affecting an amino acid close to the cysteine (145) residue at the active site of MGMT. In vitro assays did not reveal any influence of the amino acid substitutions on the activity of the protein on methylated DNA substrate, however, the $Val^{143}$ variant was more resistant to inactivation by the MGMT inactivator $O^6$-(4-bromothenyl)guanine. Finally, the relationship between alleles at the two sites and MGMT expression levels allows the prediction of MGMT activity in individuals according to their genotype and we report the results from a case-control series suggesting a link between MGMT activity and lung cancer risk.

© 2004 Cancer Research UK

### P155

### COMPLEX PHENOTYPES ASSOCIATED WITH URACIL IN DNA IN MAMMALS

H Nilsen [1], Q An [1], J Fitzgibbon [2], A Lister [2], T Lindahl [1]
[1] Belgium, Cancer Research UK, London Research Institutes, Clare Hall Laboratories, South Mimms, Herts, [2] United Kingdom, Cancer Research UK, Barts Medical Oncology Unit, St Bartholomew's Hospital, London

Deamination of cytosine, either of spontaneous origin or enzymatically induced, leads to the generation of U:G mismatches in DNA, that upon replication will lead to the formation of a C to T transition mutation in one daughter strand. On the other hand, misincorporation of dUMP into DNA during semiconservative replication leads to the formation of U:A base pairs, lesions that are neither cytotoxic nor mutagenic. We have observed an increased steady-state level of uracil in DNA in gene-targeted mice deficient in the Ung uracil-DNA glycosylase, so Ung is the major enzyme removing uracil residues from U:A base pairs. The absence of a generalised mutator phenotype in *ung*$^{-/-}$ mice indicated that complementary uracil-DNA glycosylases were responsible for the removal of uracil from U:G base pairs resulting from spontaneous deamination of cytosine in the general genome. However, Ung has recently been shown to have a specific role in processing U:G base pairs generated by enzymatic deamination of cytosine by AID (activation-induced deaminase) during class-switch recombination and somatic hypermutation. We have found that aging Ung-deficient mice develop B-cell lymphomas. Our data therefore suggest that Ung is a murine tumour suppressor gene. We are analysing genome instability in Ung-deficient murine tumours to attempt to unravel the molecular mechanisms underlying lymphomagenesis in *ung*$^{-/-}$ mice. We are also extending the work to human tumour material in order to clarify if there is an occasional contribution of UNG-deficiency in human B-cell lymphomas.

### P156

### THE ROLE OF TUMOUR ENDOTHELIAL MARKER-8 (TEM-8) IN HUMAN COLON CANCER AND ITS CORRELATION WITH PROGNOSIS

Khaled Rmali , M CA Puntis , Wen G Janig
United Kingdom, Department of Surgery, University of Wales College of Medicine, Cardiff

**Background:** TEM-8 is considered to be amongst several genes elevated only in human tumour endothelium. However, little is known about its role in solid tumours including colon cancer. This study sought to examine the levels of expression for TEM-8 in human colon cancer and correlate it with tumour prognosis.
Methodology:
Cancer and normal colorectal samples were obtained after surgery. RNA was extracted from frozen sections for gene amplification. The expression of TEM-8 was assessed using RT-PCR, and the quantity of their transcripts was determined using real-time-quantitative PCR (Q-RT-PCR). Due to the generation of TEM-8 antibody in our lab, for the first time the level of expression of TEM-8 examined at protein level using Western blotting. Blood vessels and colon tissues (normal and cancer) were stained immunohistochemically using anti-vonWillebrand Factor and anti-TEM-8 antibodies respectively. Statistical analysis carried out using student *t* test.
Results:
TEM-8 expression was significantly higher in the tumour tissues compared to the normal colon mucosa (p=0.001), the transcripts of TEM-8 were higher in patients with Duke C (p=0.04 vs Duke A). Malignant cells in tumour tissues stained strongly positive to TEM-8 compared with the epithelial cells in normal colon tissues, and a highly elevated number of strongly stained vessels that are positive to TEM-8, but not for vonWillebrand Factor.
Conclusion:
The aberrant expression of TEM-8 in invasive colon cancer could have significant prognostic and therapeutic implications. Measuring this molecule in colon cancer tissues may provide, for the first time, important molecular indicators of tumour differentiation, aggressiveness and prognosis.

### P157

### SODIUM SALICYLATE INHIBITS INFLAMMATION INDUCED INVASION AND MIGRATION OF HUMAN MELANOMA CELLS

E Katerinaki [1], R Lalla [1], JW Haycock [1], PC Lorigan [2], S MacNeil [1]
[1] Department of Engineering Materials, University of Sheffield, Sheffield, United Kingdom, [2] Department of Medical Oncology, Christie Hospital, Manchester, United Kingdom

We have previously shown that TNF-alpha upregulates human melanoma cell integrin expression, migration and invasion *in vitro* [1, 2]. The aim of the current study was to investigate the effect of the non-steroidal anti-inflammatory agent sodium salicylate on TNF-alpha induced activation of the transcription factor NFkappaB and on migration and invasion of melanoma cells.
HBL human melanoma cells were pre-incubated with sodium salicylate at concentrations ranging between 0.1 and 5 mM prior to stimulation with TNF-alpha at 200 units/ml for 24 hours. NFkappaB activation was measured using an assay that detects changes in the expression of a luciferase reporter gene under the direct control of NFkappaB transcriptional activity. The effect of sodium salicylate (5 mM) and TNF-alpha (200 units/ml) on HBL cell invasion over 20 hours and migration over 24 hours were studied using fibronectin invasion and "scratch wound" migration models *in vitro* as described previously [2].
Sodium salicylate inhibited TNF-alpha stimulated NFkappaB activation in melanoma cells in a concentration-dependent manner. The maximum effect observed was at a salicylate concentration of 5 mM that decreased TNF-alpha stimulated activation of NFkappaB by 32% (n=3, p<0.01). Time course experiments showed that this effect was achieved with pre-incubation times as short as 15 minutes (28% inhibition). In functional assays TNF-alpha stimulated HBL cell migration on tissue culture polystyrene and invasion through fibronectin were completely inhibited by sodium salicylate.
In conclusion, sodium salicylate effectively inhibited TNF-alpha induced upregulation of gene transcriptional activity, *in vitro* migration and invasion in human melanoma cells, indicating that non-steroidal anti-inflammatory drugs may be a useful therapeutic approach to oppose inflammation induced melanoma invasion and metastasis *in vivo*.

(1) Zhu N *et al*. *J Invest Dermatol* 2002; 119:1165-1171
(2) Katerinaki E *et al*. *Br J Cancer* 2003; 89: 1123-1129

### P157:1

### 5T4 ONCOFOETAL ANTIGEN HETEROLOGOUS PRIME-BOOST WITH E1 DELETED ADENOVIRUS AND DENDRITIC CELLS INDUCES SPECIFIC CD8$^+$ T CELLS AND TUMOUR IMMUNITY

S Ali , K Mulryan , T Taher , P.L Stern
United Kingdom, Paterson institute of Cancer Research, Manchester

5T4 is an oncofoetal antigen, discovered by looking for molecules shared by human trophoblast and cancer cells. It is expressed by a wide spectrum of tumours but with restricted normal tissue expression. In colorectal, gastric and ovarian cancer 5T4 tumour expression is associated with poor clinical outcome. The restricted adult tissue but high frequency and cell surface expression by multiple carcinoma types makes 5T4 antigen an attractive target for both antibody and cell mediated immunotherapies. Early tumour studies using MVA as a gene therapy vector have established the potential for 5T4 as an immunotherapeutic target and have led to its translation into clinical trial (Mulryan et al., Mol Cancer Ther 2002; 1: 1129-37). We have now shown that E1 deleted recombinant adenoviruses expressing 5T4 are effective gene therapy vectors for the induction of specific CD8 T cells. A limitation to the use of any viral vector would be the presence of pre-existing neutralising antibodies as well as generation of anti-viral cellular immunity. Production of a maximal immune response can be facilitated by a prime heterologous boost approach to vaccination. We have developed 5T4 retrovirally transduced DC based carriers and used them in combination with the adenoviral vectors. We show that such heterologous prime boost regimens are highly efficient in increasing the 5T4-specific CD8$^+$ T cell response. They provide increased survival of mice from tumour growth in both protection and active therapy assays. We also show that the order of the heterologous prime –boost immunisations can influence long term tumour immunity.

© 2004 Cancer Research UK

### P157:2

**DEVELOPMENT OF A CANCER VACCINE TARGETING COMPLEMENT REGULATORY PROTEIN CD55**

RG Bradley , JM Ramage , R Metheringham , I Spendlove , LG Durrant
United Kingdom, University of Nottingham, Nottingham

CD55 is one of several complement regulatory proteins (CRP's) involved in protection from autologous complement 'bystander' attack. Expression of the CRP's has been shown to be deregulated in a variety of tumours with CD55 being up-regulated up to 100 times normal levels. This has been identified as a significant mechanism for evading immune effectors by tumours and their microenvironment. A cancer vaccine specifically targeting this associated antigen could be used to promote existing humoral responses and to induce cytotoxic cellular mechanisms vital for tumour therapy.
Utilising known algorithms for the prediction of HLA-A2 epitopes we identified sequences from within CD55 and modified known anchor residues to enhance binding to MHC molecules. These $CD8^+$ peptides were synthesised and epitope modification was confirmed via MHC stabilisation assay on T2 cells. These enhanced epitopes have been incorporated into CD55 (DNA)-Fc constructs and used with Helios Gene Gun technology to immunise HLA-A2 transgenic mice. This strategy enabled in vivo expression of CD55-Fc protein containing our modified residues. One of these epitope enhanced constructs generated a frequency of 1 cell in 5 x $10^3$ IFNγ secreting cells specific to both native and modified peptides as demonstrated by ELISPOT analysis. These findings were further elucidated by the presence of epitope specific CTL responses to T2 targets pulsed with both modified and native epitopes as assessed by $^{51}$Cr release. Our findings indicate that modification of anchor residues can enhance epitope specific responses that recognise native sequences, indicating that the target epitope is not lost during modification.
These results suggest the presence of a T cell repertoire specific for CD55 and the efficacy of this vaccine will be assessed in tumour models. This strategy provides a novel approach for the development of a cancer vaccine.

### P157:3

**PALMITOYLATION OF MT1-MMP AT CYSTEINE574 IN THE CYTOPLASMIC TAIL FACILITATES ITS INTERNALISATION AND MODULATE CELL MIGRATION**

N Anilkumer , T Uekita , J Couchman , M Seiki , H Nagase , Y Itoh
[1] United Kingdom, Imperial College London, London,
[2] United Kingdom, University of Tokyo, Tokyo

Membrane type 1 matrix metalloproteinase (MT1-MMP) is a type 1 transmembrane proteinase that promotes cell migration and invasion by degrading components of the extracellular matrix and processing various membrane proteins. MT1-MMP has a short cytoplasmic tail of 20 amino acids which has been shown to direct internalisation of the enzyme via incorporation into clathrin-coated pits. This occurs through interaction of the Leu-Leu-Tyr$^{573}$ motif of the cytoplasmic domain with the μ2 subunit of Adapter Protein-2 (AP-2). Immediately downstream of the Leu-Leu-Tyr$^{573}$ motif, there is a Cys$^{574}$ which is a potential palmitoylation site as it is located 12 amino acids away from the membrane proximal region. Thus in this study, we investigated the palmitoylation of MT1-MMP and its effects on the biological function of the enzyme. Palmitoylation of MT1-MMP was studied using metabolic labelling of transiently transfected COS-7 cells with [$^3$H]palmitic acid, followed by immuno-precipitation and autoradiography. The data indicated that MT1-MMP is palmitoylated at Cys$^{574}$ in the cytoplasmic domain as Cys574Ala or Ser mutant was not palmytoylated. Palmitoylation of the enzyme was found to be critical for its rapid internalisation, though it did not appear to affect the localisation of MT1-MMP to lipid rafts. Analysis of further mutant molecules suggested that palmitoylation facilitates the clathrin-mediated internalisation of the enzyme, by positioning the Leu-Leu-Tyr$^{573}$ sequence closer to the plasma membrane thereby facilitating its interaction with AP-2 and subsequent incorporation into clathrin-coated pits.

### P157:4

**ONCOSTATIN M-SPECIFIC SIGNALLING EVENTS INVOLVED IN BIOLOGICAL RESPONSES OF T47D BREAST TUMOUR CELLS**

N Underhill-Day , JK Heath
United Kingdom, University of Birmingham, Birmingham

Oncostatin M (OSM) is a member of the interleukin-6 (IL-6) cytokine family which use a common signal-transducing receptor subunit, gp130. OSM acts on target cells through interactions with either the gp130/LIFRα receptor complex, also utilised by LIF, or the gp130/OSMRβ receptor heterocomplex, specific to OSM. Formation of these high affinity receptor complexes results in activation of JAK/STAT and MAPK signalling pathways.
Oncostatin M is of particular interest due to its potent ability to initiate differentiation and inhibit the growth of various human, tumour-derived cell lines. However, it is unclear to what extent the different receptor complexes contribute to these biological outcomes. The molecular interactions that underlie OSM-induced cytostatic and morphological effects have not yet been resolved.
In this study we used breast tumour-derived cell lines to compare STAT & MAPK activation and downstream biological effects resulting from ligand-induced stimulation of the representative hetero- and homodimeric receptor complexes (i.e. gp130/LIFRα, gp130/OSMRβ and gp130/gp130). We found, through LIFRα antagonism in conjunction with OSM exposure, that the potent cytostatic and morphological effects of OSM on breast tumour cell lines are mediated through the gp130/OSMRβ complex and not the gp130/LIFRα complex. Stimulation with OSM, LIF and IL-6 initiated differential molecular profiles of STAT and MAPK pathway activation. In particular, OSM proved to be a potent activator of JNK/SAPK and JNK-activated transcription factors. Stimulation with OSM in the presence of an inhibitor of JNK serine/threonine kinase blocked OSM-induced morphological changes revealing an important role for JNK/SAPK in OSM-induced cytostatic specificity.

### P157:5

**EPIDERMAL GROWTH FACTOR RECEPTOR TRAFFICKING BY SYNTAXIN**

S Saxena [1], R Kharidia [1], S Cemeller [1], M Singh [1], S Kaur [1], A Saxena [2]
[1] United States, Stevens Institute of Technology, Hoboken,
[2] United States, Robertwood Johnson Medical School, New Brunswick

Epidermal growth factor (EGF) receptors (EGFRs) belongs to tyrosine kinase receptor family and are involved in signal transduction and the regulation of cellular proliferation and differentiation of variety of cancers, including those of the lung, breast and ovary. Although members of the EGF receptor family are found predominantly at the cell surface, these receptors also undergo constant cycling between the plasma membrane and the endosomal compartment. Syntaxins and vesicle-associated membrane protein (VAMP) families, also known as soluble N-ethylmaleimide-sensitive factor (NSF) attachment protein receptors (SNARE), have been implicated in mediating membrane fusion and may play a role in determining the specificity of vesicular trafficking. Syntaxins play a general role in membrane trafficking by acting as target membrane-specific receptors for transport vesicles. Our studies suggest that EGF receptor interacts with syntaxin, a t-SNARE through protein-protein interaction. We used syntaxin antibody to pull down EGF receptor from pancreatic cancer cells, miapaca, induced with epidermal growth factor. Alternatively, EGF receptor antibody pulled down syntaxin and concomitant SNARE proteins involved in vesicular trafficking from cell homogenates. EGF receptor expression can be blocked by over-expressing ΔC syntaxin construct lacking membrane binding domain. Moreover, syntaxin over-expression modulates cellular proliferation. These data indicate towards the role of syntaxin in regulation of EGF receptor trafficking and possibly receptor signaling.

© 2004 Cancer Research UK

### P157:6

### DEVELOPMENT OF A PREDICTIVE RISK ASSESSMENT MODEL FOR DIET RELATED CHEMICALS USING COLONIC CRYPT STEM CELL MUTATION INDICES

ET Donnelly [1], H Bardwell [2], GA Thomas [2], ED Williams [2], M Hoper [1], P Crowe [1], WG McCluggage [3], M Stevenson [4], DH Philips [5], A Hewer [5], M Osborne [5], FC Campbell [1]
[1] United States, Surgery, Cancer Centre, The Queen's University of Belfast, Belfast, [2] United Kingdom, Strangeways Research Laboratories, University of Cambridge, Cambridge, [3] United Kingdom, Pathology, School of Medicine, The Queen's University of Belfast, Belfast, [4] United Kingdom, Epidemiology, School of Medicine, The Queen's University of Belfast, Belfast, [5] United Kingdom, Section of Molecular Carcinogenesis, Institute of Cancer Research, Surrey

**Background:** Humans are exposed to complex mixtures of dietary chemicals that may be implicated in colorectal cancer (CRC) but risk assessment is hampered by the lack of a mechanistically based predictive model. We test the hypothesis that colonic crypt stem cell mutation indices (CCSCMI) including total mutation load, mutant patch formation and mutation frequency, may provide the scientific basis for a novel model.
**Methods:** N-methyl-N-nitrosourea (MNU) and undegraded lambda carrageenan (λCgN) were tested as model chemicals alone or within a mixture, upon CCSCMI in 90 female Balb/c mice. The predictive power of CCSCMI was tested against early tumour development, represented by aberrant crypt foci (ACF), at 20 weeks after the start of treatment.
**Results:** Combined λCgN/MNU treatment regimens were associated with greater total crypt stem cell mutation load ($p<0.01$), greater mutant patch formation ($p<0.05$), greater numbers of large mutant patches (≥ 3 mutant crypts; $p=0.002$), greater number ($p<0.001$) and size ($p<0.01$) of aberrant crypt foci, than MNU alone. Linear correlations were observed between total crypt stem cell mutation load, number ($r=0.732$; $p<0.01$) and size ($r = 0.84$; $p<0.01$) of aberrant crypt foci, in murine colon.
**Conclusions:** This study has developed and tested a risk assessment model based on CCSCMI that appears suitable for risk assessment of dietary chemical mixtures. The model reflects exposure patterns, improves understanding of interactive mechanisms and predicts early tumour formation. This model may be useful for evaluation of chemopreventive therapy.

### P157:7

### CELL CYCLE DEPENDENT CHROMATIN PROTEOMICS: IDENTIFICATION AND PRELIMINARY CHARACTERISATION OF NOVEL CHROMOSOME BINDING PROTEINS

I.M Porter [1], G.A Khoudoli [1], J.S Andersen [2], J.J Blow [1], J.R Swedlow [1]
[1] United Kingdom, University of Dundee, Dundee, [2] United Kingdom, University of Southern Denmark, Odense

Many important events of the cell cycle directly involve chromatin and chromosomes. These include chromosome replication, nuclear assembly and reassembly, chromosome condensation and apoptosis. Some of the protein factors involved in these processes are known, but a full analysis of their cell-cycle dependent chromatin association has not been achieved. Many more factors likely remain to be identified and studied.
We have examined the proteome of chromatin and chromosomes at specific stages of the cell cycle. Interphase chromatin and mitotic chromosomes were generated *in vitro* by incubating sperm nuclei in Xenopus egg cytoplasmic extracts. After isolation of chromatin and chromosomes, associated proteins were eluted and soluble protein fractions were subjected to LC/LC-MS/MS or 2D gel electrophoretic analysis.
LC/LC-MS/MS analysis identified over 450 chromatin bound proteins, with 150 common to mitotic chromosomes and interphase chromatin. Of 68 proteins of unknown function 22 were identified as orthologues of human proteins. We have developed an siRNA-based microscopic screen to characterise the properties of these proteins and have validated the approach with six candidate proteins. When depleted from cells, each of these proteins causes significant defects in mitotic progression. In addition, quantitative 2D gel comparison of soluble chromatin fractions isolated at different stages of G1 and S-phase has revealed approximately 50 proteins which have altered affinity for chromatin throughout DNA replication, including a number of previously uncharacterised proteins. Functional analysis of these proteins is underway.

### P157:8

### ETS GENE, PEA3 COOPERATES WITH Β-CATENIN-LEF-1 AND C-JUN IN REGULATION OF OSTEOPONTIN TRANSCRIPTION

M El-Tanani [1], A Platt-Higgins [2], P.S Rudland [2], F.C Campbell [1]
[1] Denmark, Surgery, Cancer Centre, The Queen's University of Belfast, Belfast, [2] United Kingdom, Cancer and Polio Research Laboratories, School of Biological Sciences, University of Liverpool, Liverpool

**Introduction:** Upregulation of osteopontin (OPN) is a central event of neoplastic transformation, colorectal cancer (CRC) metastasis and prognosis. OPN is not typically mutated and molecular mechanisms responsible for its upregulation are unclear. The OPN promoter contains binding motifs for β-catenin-Tcf-lef-1, Ets and AP-1 which are commonly disturbed during colorectal tumorigenesis.
**Aim:** We test the hypothesis that β-catenin-Tcf-lef-1, Ets and AP-1 signaling are implicated in combinatorial dysregulation of OPN transcription.
**Methods:** Rama 37 epithelial cells were transiently co-transfected with wild type and mutant OPN promoter-luciferase reporter constructs mutated at Ets, Tcf and AP-1 binding domains. Effects of Ets factors, Ets-1, Ets-2, ERM and PEA3, as well as β-catenin, lef-1 and c-jun upon OPN-promoter luciferase activity and endogenous OPN expression were assessed.
**Results:** β-catenin, Lef-1, Ets transcription factors and the AP-1 protein c-jun, each weakly enhanced luciferase expression from a OPN promoter-luciferase reporter construct. OPN promoter responsiveness to β-catenin and Lef-1, however, was considerably enhanced by Ets transcription factors including Ets-1, Ets-2, ERM and particularly PEA3. PEA3 also enhanced promoter responsiveness to the AP-1 protein, c-jun. Co-transfection of cells with β-catenin, Lef-1, PEA3 and c-jun in combination increased luciferase expression by up to 280 fold and induced expression of endogenous OPN.
**Conclusions:** This study shows that β-catenin/Lef-1, Ets and AP-1 transcription factors can cooperate in stimulating transcription of OPN. These transcription factors may be responsible for OPN over-expression which is implicated neoplastic transformation CRC progression and metastasis.

### P157:9

### IDENTIFICATION OF THE COREGULATOR (S) THAT INTERACTS WITH THE OSTEOPONTIN PROTEIN IN THE BREAST CANCER CELLS

D Jin , FC Campbell , M El-Tanani
United Kingdom, Surgery, Cancer Centre, The Queen's University of Belfast, Belfast

Introduction:
Osteopontin (OPN) is a multifunctional protein implicated in mammary development, neoplastic change and metastasis. OPN is a target gene for beta-catenin-Tcf signaling, which is commonly disturbed during mammary oncogenesis but the understanding of OPN regulation is incomplete. Coactivators and corepressors of OPN have not identified, little is known of its mechanism of action.
Methods:
Bacterial two-hybrid system has been used to isolate the cDNA coding for a protein (s) (target proteins) that binds specifically to the osteopontin (bait protein). The mammalian two-hybrid system was performed for protein-protein interaction confirmation.
Results:
cDNAs codes for five novel transcription factors (target genes) which interacted specifically with OPN, have been isolated. These five novel genes have been accepted for Genbank publication. Their accession numbers are AY562498, AY562499, AY562500, AY562501 and AY562502.
Conclusions:
This study shows novel regulatory mechanisms of OPN transcription involving 5 novel genes. These novel mechanisms may be implicated in OPN mediated neoplastic transformation.

© 2004 Cancer Research UK

### P157:10

### ACTIVATION OF HUMAN MONOCYTES VIA CD55 (DECAY ACCELERATING FACTOR)

HJ FitzGibbon , I Spendlove
United Kingdom, University of Nottingham, Nottingham

CD55 (also known as decay accelerating factor, DAF) is a glycosylpho sphatidylinositol (GPI-)linked protein which acts to limit complement deposition on cell surfaces. It is found embedded in the membranes of most serum-exposed cells as well as being overexpressed by a range of solid tumours and deposited within tumour stroma. However, tumour cell CD55 expression exceeds that required for maximal complement regulation, suggesting that the molecule may have a role beyond that of a complement regulator within the tumour environment. In support of this, studies have also reported that CD55 is able to act as a signalling molecule.

Using flow cytometry, we have shown that stimulation of CD55 on resting human monocytes, using the antibody 791T/36, results in the upregulation of a number of surface molecules involved in antigen presentation, such as the co-stimulatory molecules CD80 and CD86. The changes in expression of the molecules analysed were similar to those observed upon lipopolysaccharide (LPS)-induced monocyte activation, suggesting that stimulation of CD55 may be able to activate the cells.

To show that these results were not due to LPS contamination of 791T/36, we demonstrated an abrogation of these effects following heat inactivation of the antibody at a temperature shown to have no effect on the monocyte-activating ability of LPS.

These findings suggest that CD55 may be an important signalling molecule on antigen-presenting cells and may have a co-stimulatory role in the activation of T-cells. To investigate this further we will be assessing the cytokine profile and effector function of CD55-stimulated monocytes.

### P157:11

### TRANSFECTION OF THE CHEMOKINE RECEPTOR CXCR4 ALTERS THE BEHAVIOR OF OVARIAN CANCER CELLS *IN VITRO* AND *IN VIVO*

H Kulbe , JL Wilson , FR Balkwill
United Kingdom, Cancer Research UK, London

The aim of this study was to understand the significance of CXCR4 expression in ovarian cancer. Of 14 chemokine receptors investigated, only CXCR4 was expressed by human ovarian cancer cell lines. However, within ovarian tumor biopsies a minority of tumor cells (1-20%) expressed CXCR4 mRNA. To clarify the role of CXCR4 in ovarian cancer, we transfected the SKOV-3 cell line to produce two different SKOV-X4 clones, with high CXCR4 expression, and mock transfected lines, SKOV-M, with similar, low CXCR4 expression to the parental cell line. Stimulation of SKOV-X4 cells with CXCL12 resulted in directed migration and invasion, proliferation under sub-optimal growth conditions, increased adhesion to fibronectin and induction of TNF-α mRNA. Spheroids formed by SKOV-X4 cells were smaller than those of SKOV-M, had less well defined borders and were more spread. Mice injected with SKOV-M cells were killed between 4 and 10 weeks due to peritoneal disease, whereas those injected with SKOV-X4 survived for a median of 12 weeks. Although the volume of SKOV-X4 tumor was smaller in the abdominal cavity there were more invasive and metastatic foci at distant sites. SKOV-M tumor deposits surrounded the liver, spleen and omentum whereas SKOV-X4 deposits were widely disseminated into the mesenteric tissue, ovaries, lymph nodes, pancreas, thoracic cavity, lungs, and colon. SKOV-M tumors had better histological differentiation, lower mitotic index and did not invade tissues. SKOV-X4 tumors were highly invasive, with poor histological differentiation and higher mitotic index. Thus, enhanced CXCR4 expression results in an ovarian tumor cell with altered adhesive capacity and a more invasive phenotype.

### P157:12

### ESTABLISHING CELLULAR MODEL FOR STUDYING THE CARCINOGENESIS OF HUMAN OVARIAN EPITHELIUM

N Li , G Wilbanks , D Dafou , I Jacobs , F Balkwill , S Gayther
United States, Bart's and The London, Queen Mary's Medical School, London

Approximately 90 % of malignant ovarian tumours are epithelial and thought to arise from a single cell layer, the ovarian surface epithelium (OSE). In culture, human normal OSE cells have a very limited lifespan before they senesce, rarely progressing beyond 10 population doublings. This has restricted the use of normal ovarian surface epithelial cells (NOSE cells) for identifying molecular events *in vitro* that may contribute to malignant transformation. We have investigated the conditions for culturing human NOSE cells *in vitro* using modified medium supplemented with epidermal growth factor (EGF), hydrocortisone (HC), insulin and bovine pituitary extract (BPE) – NOSE CM medium. NOSE CM medium significantly improved the seeding and cloning efficiencies, overall cell growth and life span compared to culturing in previously established non supplemented medium. Furthermore, these improvements in cell growth in NOSE-CM occurred without detrimental effects to the epithelial morphology of the cells. Based on this optimised culture condition, we used a retroviral vector introducing hTERT, a gene encoding the functional catalytic sub-unit of telomerase, into NOSE cells and established immortal human normal ovarian surface epithelial cells (IOSE cells). The telomerase activation has been confirmed by TRAP assay and the stable telomere length has been identified. *In vitro* and *in vivo* growth patterns such as serum dependence, contact inhibition growth, anchorage dependence and tumourogenic ability in nude mice confirmed the immortality of IOSE cells. Furthermore, these IOSE cells are transformed by fractional doses of γ-ray irradiation. The malignancy of cells during the whole transformation procedure are assessed by both *in vitro* and *in vivo* assays. The transformed ovarian surface epithelial cells (TOSE cells), together with the parental immortal cells (IOSE cells) and normal primary cells (NOSE cells) lineage forms a cellular model which deliberates broad biological utilities and greatly enables the studies to determine the carcinogenesis of human ovarian surface epithelium.

### P157:13

### *IN VITRO* MODEL OF MALIGNANT TRANSFORMATION FOR THE IDENTIFICATION OF GENES IN BREAST CANCER FORMATION

D Dafou [1], NF Li [1], DA Trott [2], IJ Jacobs [1], SA Gayther [1], RF Newbold[1]
[1] United Kingdom, Barts and the Royal London School of Medicine, Queenmary University, Translational Oncology Laboratory, John Vane Science Building, EC1M 6BQ, London, [2] United Kingdom, Institute of Cancer Genetics and Pharmacogenomics, Brunel University, Uxbridge, UB8 3PH, London

Cancer is a multistep disease where by genetic and environmental factors represent important stages in the promotion, initiation and progression of the disease. Cell lines derived from tumours have been valuable tools for investigating the pathogenesis of cancer and possible therapeutic agents. However, the delineation of the early events of initiation/promotion, using such cell lines can be ambiguous. Normal human primary cells derived from the target tissue at risk for neoplastic transformation provides a novel approach that can be used to study the mechanistics of cancer progression *in vitro.*

Establishing models to investigate multistep process of malignant transformation may enable the different stages of transformation to be isolated and studied in detail. Cell lines derived from normal human primary cells can be analysed for alterations to their phenotype and gene expression among them. This includes the analysis of genetic alterations and their association with different transformation levels, including key oncogenes (c-myc, erb-2, k-ras) and tumour suppressor genes (BRCA1, BRCA2, p53) involved in breast cancer.

We have established such a model for breast cancer from primary human mammary epithelial cells (HMECs). Briefly, normal HMECs have been successfully immortalised by over-expressing the catalytic subunit of the enzyme telomerase (hTERT). The 'immortal' cell line has been subjected to fifteen fractions of ionising radiation (2 Gy per fraction). During the irradiation process cell lines were established with ever increasing levels of transformation and quantified at the cellular (ie. anchorage independence, foci formation), genetic (SKY-FISH) and molecular genetic level (real –time RT-PCR). cDNA expression microarrays were used to compare and identify patterns of gene expression of HMECs irradiated with 14 and 20 Gy dose of irradiation respectively. This has lead to the identification of genomic changes that may be critical during breast carcinogenesis. These experiments, show the fundamental importance of the use of *in vitro* human cell systems for the identification of molecular markers in breast cancer formation.

© 2004 Cancer Research UK

### P158

### VALIDATION OF PHARMACODYNAMIC ASSAYS TO EVALUATE THE CLINICAL EFFICACY OF AN ANTISENSE TARGETED TO THE XIAP INHIBITOR OF APOPTOSIS

J Cummings [1], T H Ward [1], E LaCasse [2], M Dawson [1], O Denneny [1], D McManus [2], J Durkin [2], M Ranson [3], C Dive [1]
[1] United Kingdom, Clinical and Experimental Pharmacology, Paterson Institute for Cancer Research, Christie Hospital NHS Trust, Wilmslow Road, Manchester, [2] United Kingdom, Aegera Oncology Inc., Ottawa, [3] Canada, Department of Medical Oncology, Christie Hospital NHS Trust, Wilmslow Road, Manchester

XIAP is a potent endogenous caspase inhibitor frequently overexpressed in human tumours. Recently, a 19-mer second generation antisense oligonucleotide targeting XIAP has been approved for a Phase I Trial. Validation strategies and performance data on 3 different pharmacodynamic (PD) assays will be presented that will be utilised as endpoints during this trial. To facilitate validation, we employed a genetically-engineered cell line (MDA XG4) stably expressing a RNAi to silence XIAP as a negative control. Immunohistochemistry (IHC) of XIAP in tumour biopsies was validated using sections prepared from fixed pellets/tissues derived from XG4 cells and panel of cancer cell lines that represent a 3-4-fold dynamic range of XIAP protein expression. These calibrators defined staining intensity levels against which the patient samples will be assessed. Studies were performed on within-day and between-day precision and on the stability of XIAP in the fixed sections. The M30-Apoptosense™ Elisa detects a caspase cleaved fragment of cytokeratin 18 that is released into the circulation after apoptotic cell death and is believed to represent a plasma surrogate marker enabling a quantitative assessment of apoptosis occurring in the patient's tumour. Validation of this technique focussed on generating an independent positive quality control to perform measurements on precision, kit-to-kit QC, and stability studies. Precision data obtained using this control ranged from 3.6-6.7%. Kit to kit variability was less than 10%, while samples stored at $-80^{0}$C were demonstrated to be stable for at least two months. Western blotting of XIAP was validated using replicate frozen pellets of 1 negative and 2 positive control cell lines. XIAP was quantitated by densitometry as a ratio to GAPDH and by reference to a calibration curve constructed using recombinant XIAP protein. To determine the linearity and the dynamic range of the assay, increasing amounts of recombinant GST-XIAP or of the BIR3-RING fragment were added to lysates of the null cell line.

### P159

### DEVELOPMENT OF A CELL FREE SYSTEM TO IDENTIFY MOLECULAR CONNECTIONS BETWEEN DNA DAMAGE AND APOPTOSIS IN CEM T LYMPHOMA CELLS

I Moteiro dos Santos Pires [1], SP JAckson [2], C Dive [1]
[1] United Kingdom, Paterson Institute for CAncer Research, Manchester, [2] United Kingdom, Wellcome Trust/CAncer Research UK Gurdon Institute, Cambridge

Resistance to DNA damaging drugs is a formidable obstacle to chemotherapy approaches to disease treatment. This resistance is due, at least in part to an uncoupling of DNA damage signals to the engagement of apoptotic cell death. A cell free system has been developed in which isolated naïve mitochondria are incubated with a 'drug activated' cytosolic fraction in order to identify DNA damage signals that are conveyed to the apoptotic machinery at the mitochondrial surface. Treatment with the UV mimetic 4-NQO (10 μM) resulted in the activation of CHK1 by 15 min whereas CHK2 was not activated until 30 min. 4-NQO treatment resulted in 20% and 50% apoptotic cells at 4h and 8h respectively. A detailed examination of caspase activity was performed using a fluorogenic assay in order to define a time point after drug treatment at which DNA damage had been recognised and signalled (as indicated by activation of CHK2 or CHK1) and the damage signals generated could release cytochrome c upstream of caspase activation. The earliest time point at which cytochrome c releasing signals, generated by 4NQO mediated DNA damage, were present in cytosolic fractions was 4h. Cell free experiments were performed in the presence and absence of the pan caspase inhibitor ZVAD (100μM). Cytochrome c was released from naïve mitochondria incubated with cytosols from cells treated for 4h with 4NQO regardless of caspase inhibition by ZVAD. The roles of Bax and Bak are now being investigated in this system as well as an examination of the levels and subcellular location of BH-3 only Bcl-2 family members. 4-NQO activated, ZVAD treated cytosols will now be fractionated and the fractions applied to naïve mitochondria in order to identify the DNA damage mediated, caspase independent cytochrome c releasing signalling molecules.

### P160

### LYMPHOCYTE DEATH BY CATIONIC POLYMERS: A ROLE FOR MITOCHONDRION AND IMPLICATIONS IN GENE THERAPY

JC Murray [1], SM Moghimi [2], AC Hunter [2], P Symonds [1], G Debska [3], A Szewczyk [3]
[1] United Kingdom, Wolfson Digestive Diseases Centre, University Hospital, Nottingham, [2] United Kingdom, School of Pharmacy and Biomolecular Sciences, University of Brighton, Brighton, [3] United Kingdom, Nencki Institute of Experimental Biology, Polish Academy of Sciences, Warsaw

A wide range of synthetic polycations in linear, branched, or dendrimer form have been used to condense DNA into structures amenable to cellular internalization via endocytosis. Polycations can destabilize endosomal membranes or act as proton sponges; they buffer the low pH in the endosomes and potentially induce membrane rupture, resulting in the release of polycation/DNA complex into the cytoplasm. The polycationic nature of the gene-delivery vehicles can induce cytotoxicity, but the mechanisms are poorly understood. We studied the effect of a number of commonly used polycations on mitochondrial functions in isolated mitochondria from rat liver as well as directly in Jurkat cells. Upon permeabilization or rupture of the outer mitochondrial membrane, cytochrome *c* binds to Apaf-1, leading to allosteric activation of pro-caspase-9. This in turn proteolytically activates caspase-3, one of the principal proteases that participates in the execution of cell death. A decrease in mitochondrial membrane potential ($\Delta\varphi$) due to permeability transition is also an early event in several types of apoptosis. We have demonstrated that at very low concentrations, polycations can affect mitochondrial respiration and $\Delta\varphi$; these events were followed by cytochrome *c* release from mitochondrial intermembrane in mitochondrial suspensions and in Jurkat cells. Changes in mitochondrial $\Delta\varphi$ in Jurkat cells were confirmed by the Mitosensor test. Detection of phosphatidylserine translocation to the cell surface and proteolytic activation of caspase-3 by apoptosome further confirmed activation of apoptosis in Jurkat cells. These observations provide a molecular explanation for previously reported immediate or delayed cytotoxicity following gene transfer with polycations. The results from this study may help to design novel materials with high transfection efficiencies suitable for clinical gene therapy.

### P161

### ETOPOSIDE INDUCED P53-DEPENDENT GENE EXPRESSION IN COLON CANCER: ANALYSIS OF BIRC3, A CANDIDATE GENE

A. Davies , A.V. McNamara , A.J.M. Watson , J.R. Jenkins
United Kingdom, University of Liverpool, Liverpool

**Background:** Loss of p53 tumour suppressor function is common to most cancer types; and is a late stage event in colon cancer tumourgensis. Etoposide (VP16) is a cytotoxic agent whose target is topoisomerase-II, an enzyme vital for successful DNA replication. Colon cancer responds poorly to Vepesid (VP16) treatment and induces toxic side effects; including the significant risk of secondary leukaemia. The aim of this study was to identify novel p53-dependent genes involved in response to VP16 treatment.
**Methods:** Array Analysis of VP16 treated (50μM; 24hrs) human colon adenoma HCT116 (p53 isogenic pair) cell lines was performed, and mRNA expression changes confirmed by Real Time PCR (qPCR). Protein expression was investigated by western blotting (WB) and immunocytofluorescence (ICF). Small interfering RNAs (siRNA) were designed to create a transient BIRC3 "knock-down" cell type for clonogenic assays.
**Results:** The Array Analysis identified a member of the Inhibitor of Apoptosis (IAP) family, Baculoviral IAP Repeat (BIRC3), which was confirmed by qPCR. This gene exhibited a p53-dependent increase in mRNA and protein (WB) expression following VP16 treatment. ICF showed a perinuclear cellular distribution. In a BIRC3 siRNA "knock-down" experiment on HCT116 cells, following treatment with VP16, an increase in cell killing of 30% was observed when compared to the mock-transfected cells.
**Conclusion:** BIRC3 is a, VP16 responsive, p53-dependent gene in the human HCT116 colon adenoma cell line. The increased expression of the Birc3 protein could be a contributory factor to an induced apoptotic resistance in colon cancer. Other research groups have also proposed that deregulation of another IAP family member, Survivin, as a contributor towards the progression of colon cancer.

© 2004 Cancer Research UK

### P162

### 4-HYDROXY(PHENYL)RETINAMIDE INDUCES APOPTOSIS IN THE EWING'S SARCOMA FAMILY TUMOURS (ESFT) THROUGH A P38$^{MAPK}$ DEPENDENT PATHWAY

SS Myatt, SA Burchill
United Kingdom, Candlelighter's Research Laboratory, Leeds

4-hydroxy(phenyl)retinamide (4-HPR) is a promising anti-cancer agent, inducing cell death in a wide range of cancer cell types and with low toxicity in paediatric phase 1 clinical trials (Cancer Lett. (2003) 197(1-2):185-92). In this study the effect of 4-HPR on viable (trypan blue exclusion assay) and apoptotic (annexin V/propidium iodide) cell number in six ESFT cells lines has been evaluated. The role of p38$^{MAPK}$ in the induction of cell death by 4-HPR has been investigated using Western blots and inhibitors of p38$^{MAPK}$ signalling (SB202190).
4-HPR (0.75-10 μM) induced apoptosis in 6/6 ESFT cell lines within 24h exposure in a dose-dependent manner. PARP cleavage was observed after 8h treatment of TC-32 cells with 4-HPR (1.5 μM). ESFT TC-32 cells were more sensitive to 4-HPR-induced death than the neuroblastoma SY5Y cell line; IC50 0.95μM and 5.3μM respectively. This suggests a novel mechanism of 4-HPR action in ESFT.
Consistent with previous studies in other cancer cell types, a dose-dependent increase in reactive oxygen species (ROS) was observed in TC-32 cells following treatment with 4-HPR. There was a 6-fold increase in ROS after 1h exposure to 4-HPR, compared to a 3-fold increase in SY5Y cells. Pre-treatment of TC-32 cells with the anti-oxidant ascorbic acid (100 μM, 1 h) prevented the accumulation of ROS and rescued 100% of TC-32 cells from 4-HPR (1.5 μM) induced cell death. p38$^{MAPK}$ was phosphorylated following exposure of ESFT cells to 4-HPR. Inhibition of the p38$^{MAPK}$ pathway with SB202190 (6.7 μM; 1h) rescued 52% of TC-32 cells from 4-HPR-induced death. This was accompanied by complete inhibition of PARP cleavage and partial reduction in ROS accumulation (25%).
In conclusion, 4-HPR induces apoptosis in all ESFT cells studied. Activation of p38$^{MAPK}$ appears to play a role in 4-HPR-induced death in ESFT. This may in part explain the increased sensitivity of ESFT to 4-HPR compared to neuroblastoma cells.

### P163

### DOWNREGULATION OF BCL-XL EXPRESSION USING SIRNA SENSITISES COLON CANCER CELLS TO TOPOISOMERASE I INHIBITOR SN-38

ML Hua, P Kang, S Guichard, DI jodrell
University of Edinburgh, Edinburgh, United Kingdom

Apoptosis is the prevalent form of programmed cell death and overexpression of anti-apoptotic proteins like Bcl-2 and bcl-xl has been linked to resistance to chemotherapeutic agents. RNA interference using small interfering RNA (siRNA) has recently been developed to specifically silence genes. We designed 4 different oligonucleotides (#1, #2, #3 and #4) inserted in pSUPER-Neo-GFP vector to express short hairpin RNA sequences targeting 4 different regions of bcl-xl mRNA. We transfected 2 colon cancer cell lines, HCT-116 and HT-29 cells with the 4 different constructs as well as the empty vector. We evaluated the transfection efficiency monitoring the GFP expression using fluorescence microscopy. HT-29 cells had very low transfection efficiency so we selected positive clones (geneticin-resistant) by growing cells in medium containing 1000 μg/ml of geneticin. The growth rates of the different isogenic cell lines stably transfected were similar with doubling times ranging from 16 to 18h. We determined the level of bcl-xl expression in the different clones by real-time RT-PCR using 2 different sets of primers. 18S rRNA expression was used as internal standard. In HCT-116 cells, the level of bcl-xl expression normalized with 18S expression were $1 \pm 0.1$, $1 \pm 0.2$, $0.6 \pm 0.1$, $0.7 \pm 0.1$, $0.5 \pm 0.07$ and $0.5 \pm 0.15$ in HCT-116 parental cells, cells expressing the empty vector, #1, #2, #3 and #4, respectively so 2 out of 4 constructs had a bcl-xl expression significantly lower than cells transfected with the empty vector. We determined the growth inhibition of the topoisomerase I inhibitor SN-38 in the different isogenic cell lines using the sulforhodamine B assay. The IC50s using 24h-exposure were $4 \pm 0.03$, $4 \pm 0.03$, $2.8 \pm 0.04$, $3.2 \pm 0.3$, $1.8 \pm 0.07$ and $2.2 \pm 0.07$ nM for HCT-116 parental, expressing the empty vector controls, #1, #2, #3 and #4, respectively. We plan to extend these results to HT-29 cells and to investigate if the decrease in bcl-xl RNA levels is correlated with an increase in apoptosis after exposure to SN-38.

### P164

### IMMUNOHISTOCHEMICAL DETECTION OF APOPTOTIC MARKERS IN GASTRIC CANCER

L Smith, HK Berrieman, SL O'Kane, A Campbell, A Maraveyas, L Cawkwell
PGMI, University of Hull, Hull, United Kingdom

Gastric cancer is the second most common malignancy worldwide and prognosis remains poor. Treatment involves the administration of chemotherapeutic agents, which act to initiate apoptosis by inducing irreparable DNA damage. Unfortunately, the objective response rate remains low and such treatment, if not beneficial for the patient, causes needless toxicity and undesirable side effects. Effective treatment is hampered by the tumours ability to exhibit various mechanisms of drug resistance. Alterations in the expression of apoptosis-related proteins may alter response to chemotherapy.
Immunohistochemistry was exploited in this pilot study to examine a comprehensive panel of apoptotic biomarkers in 21 cases of gastric cancer. This preliminary study was essential; biopsy samples taken on diagnosis (pre-treatment) are small and not enough tissue is available to test the entire panel of antibodies for heterogeneous expression. The biomarkers selected for analysis were p53, p21, Fas, Bcl-2, Bax, Bcl-xL, Bid, Bim, Bad and Bak. Aside from the well studied proteins, such as p53 and Bcl-2, few of the apoptotic markers used in this study have previously been analysed in relation to gastric cancer.
The expression of Bim, Bcl-xL and Bak was high (>75% of tumours positive), the expression of Fas, Bax, Bad, p53 and p21 was intermediate (25-75% positive) and Bid and Bcl-2 expression was low (<25%). In a further study, those biomarkers selected from this study will be applied to biopsy samples and expression will be correlated with patient response in an attempt to identify predictive markers.
It is hoped that in the future, biopsy samples taken on diagnosis will be examined for the expression of such predictive markers. The prediction of which therapeutic intervention will most likely benefit a given patient would reduce needless side effects and toxicity.

### P165

### APOPTOTIC PROTEIN EXPRESSION DIFFERS BETWEEN NON-SMALL CELL LUNG CANCER SUB-TYPES

HK Berrieman, L Smith, SL O'Kane, A Campbell, MJ Lind, L Cawkwell
PGMI, University of Hull, Hull, United Kingdom

The main form of treatment for inoperable non-small cell lung cancer (NSCLC) is chemotherapy. The status of the apoptosis pathways in NSCLC cells may therefore be crucial for treatment outcome. One major group of proteins involved in the control of apoptosis is the Bcl-2 family of proteins, but except for Bcl-2 itself, little is known about the expression of these potentially critical proteins in human cancers. A second group of proteins which may play important roles in apoptotic pathways, but which have also not been extensively studied in cancer, are the heat shock proteins (Hsps).
We have used immunohistochemistry to study the expression of Bcl-2 family proteins and Hsps in a pilot series of 41 archival NSCLC specimens. Nineteen samples were adenocarcinomas (AC) and 22 were squamous cell carcinomas (SCC). Sections were scored as positive or negative by two independent observers and associations between variables tested using Chi-square analysis.
Bax (pro-apoptotic protein), Hsp70 and Hsp27 showed high expression (>70% positive), Bak and Bid and Bad (all anti-apoptotic) showed intermediate expression (50-70% positive), and the pro-apoptotic proteins Bcl-2 and Bcl-$X_L$ showed low expression (<50% positive). Certain proteins showed statistically significant differences in expression between AC and SCC samples.
Our results, although only a pilot study, reveal significant differences in the expression of apoptosis-related proteins both between individual cases of NSCLC and also between the two main histological sub-types. Such differences could have major influence on not only the development of these tumours but also their response to chemotherapy. If such differences prove to be of clinical importance it could require that NSCLC sub-types be regarded as separate entities rather than as one disease.

© 2004 Cancer Research UK

### P165:1

**SPONTANEOUS AND DNA DAMAGE INDUCED APOPTOSIS IN A MODEL OF INTESTINAL EPITHELIAL REGENERATION**

M Hoper [1], M O'Connor [1], ET Donnelly [1], S McCullough [2], FC Campbell [1]
[1] United States, Surgery, Cancer Centre, The Queen's University of Belfast, Belfast, [2] United Kingdom, Anatomy, School of Medicine, The Queen's University of Belfast, Belfast

**Background:** Renewal tissues have a stem cell hierarchy, capacity for regeneration and are the most frequent sites of mutagen induced tumour formation. Apoptosis provides an important defence against mutagen induced cancer by elimination of irretrievably damaged cells. This study tests the hypothesis that spontaneous and DNA damage induced apoptosis are suppressed in early regeneration which may facilitate expansion of mutant epithelial clones.
**Methods:** Regeneration was induced in a model system by stem cell grafting. Stem cell aggregates from neonatal rat small intestine were implanted subcutaneously and regenerated intestinal mucosa with complete crypt/villus architecture over 3 weeks. Grafts retrieved at 7, 14 and 21 days represented early, intermediate and late stages of regeneration. To assess effects of regeneration stage on carcinogen induced apoptosis, animals were treated by N-methyl N-nitrosourea (MNU; 62.5mg/kg) vs saline control subcutaneously, 18 hours before graft retrieval at 7, 14 or 21 days. Apoptosis was assessed by the TdT-mediated dUTP nick-end labelling assay.
**Results:** Carcinogen treatment promoted apoptosis in late regenerating grafts at 3 weeks (Apoptosis - 10.6 ± 1.15% vs 6.1 ± 0.5% MNU treated vs untreated cells $p<0.05$). No evidence of an apoptosis response to mutagen exposure was found in early regeneration.
**Conclusions**: The apoptosis response to carcinogen treatment appears to be suppressed in early regeneration. Early regeneration may thus provide a window of susceptibility to carcinogen induced mutation.

### P166

***INCH*: INDUCTION CHEMOTHERAPY AND CHART.**
**A RANDOMIZED PHASE II/III OF INDUCTION CHEMOTHERAPY FOLLOWED BY CHART VERSES CHART ALONE IN PATIENTS WITH NON-SMALL CELL LUNG CANCER**

M Hatton , E Lyn , A Hodson , R Stephens
[1] Weston Park Hospital, Sheffield, United Kingdom, [2] Mount Vernon Hospital, London, United Kingdom, [3] Medical Research Council, London, United Kingdom, [4] Medical Research Council, London, United Kingdom

Introduction.
The published 2-year survival for CHART radiotherapy is comparable to that seen with induction chemotherapy and conventionally fractionated radiotherapy. Initial attempts to give induction chemotherapy with CHART have reported significant pulmonary toxicity. More recent reports have confirmed feasibility (1,2) and the experience of the UK CHART centres indicate a rate of grade 3/4/5 pneumonitis of 2 –3 %. (Personal Communication)
Aims.
To compare 3 cycle of vinorelbine/cisplatin induction chemotherapy followed by CHART radiotherapy with CHART radiotherapy alone.
The primary endpoint is overall survival; measured secondary endpoints are safety, toxicity, response rate, local control rate, progression free survival and quality of life.
Treatment.
Induction Chemotherapy: Vinorelbine (30 mgs/m$^2$) day 1 and 8; Cisplatin (80 mgs/m$^2$) on day 1 of a three weekly cycle. Three cycles.
CHART 54 Gy of thoracic irradiation in 36 fractions treating 3 times daily over 12 consecutive days.
Trial design.
A randomised, open, multi-centre, phase II/III study for patients with inoperable NSCLC, good performance status and suitable for CHART radiotherapy.
A data monitoring committee will assess feasibility and safety after 80 patients have been treated.
If an acceptable rate of toxicity is confirmed the trial will continue to recruit to 500 patients, which will give a 90% power to detect a 50% improvement in 3-year survival (from 10 to 15%).
The INCH trial will open to recruitment in spring 2004.

1) Bell V, Falk S, Gee A. Clinical Oncology 2002;14;S28.
2) Saunders MI, Rojas A, Lyn E, et al. Clinical Oncology 2002;14:352-60.

### P167

**CLINICAL TRIAL PATIENT PATHWAY: MAKING THE PATIENT JOURNEY LESS OF A TRIAL**

C May, Northern Centre for Cancer Treatment, Newcastle General Hospital, Newcastle-upon-Tyne, United Kingdom

Background: To integrate clinical trials and related research into routine care provision[1].
Objective: To define and describe a patient pathway for the enrolment of patients into a large phase III trial[2].
Setting: Large cancer centre in England.
Method: Creation of a delineated patient journey from specialist centre multi-disciplinary team meeting (MDM) to commencement of trial chemotherapy on day unit at cancer centre.
Mechanism: Acknowledging the difficulty of integrating research with routine service provision; working collaboratively, inter-professionally and inter-departmentally to create protocols and profroma; and acknowledging a clear, but compatible division of labour between research tasks and routine chemotherapy tasks within this framework.
Outcome: Patient journey: MDM to commencement of treatment 17-21 days (though not a fixed timeframe, as flexible enough to allow for patient choice[3]). Trial activities, with fully informed consent, completed within this timeframe and alongside routine treatment preparation, thus incorporating effective provision of care for patients who decline to participate in the trial. Standardised quality of care, and full participation of the patient, who is enabled to make an informed choice about her own pathway through treatment.

1. Department of Health, Sept 2000, The NHS Cancer Plan: a plan for investment a plan for reform, Topic 10
2. NHS Executive, Health Services Directive, The Manual of Cancer Service Standards, Topic 2
3. Department of Health, Sept 2000, The NHS Cancer Plan: a plan for investment a plan for reform, Topic 5

### P168

**THE ESTABLISHMENT OF A PORTFOLIO OF LIVER CANCER TRIALS WITHIN A UK CLINICAL TRIALS UNIT**

DP Brant , JM Barnwell , JA Dunn , PJ Johnson
Cancer Research UK Institute for Cancer Studies Clinical Trials Unit, Birmingham, United Kingdom

There are four main primary liver cancers, the most common being hepatocellular carcinoma and cholangiocarcinoma or biliary tract cancer. The incidence of primary liver cancer has increased over the last ten years with hepatocellular carcinoma doubling and cholangiocarcinoma increasing 10 fold.
Hepatocellular carcinoma is responsible for approximately 1500 deaths per year in the UK. The only chance of cure is surgery, with few patients being candidates. Currently there is no standard therapy for patients with unresectable or recurrent hepatocellular carcinoma. The median survival rate for these untreated patients reported as less than 2 months.
Cholangiocarcinoma is responsible for approximately 1000 deaths a year in England and Wales. The majority are advanced at presentation and of those patients that do proceed to potentially curative resection, the majority relapse with advanced disease.
The Trials Unit currently has two multicentre trials in these two common liver cancers. The hepatocellular carcinoma trial, HEP-1 is a NCRN trial funded by Cancer Research UK, and is due to start recruitment in June 2004, and the biliary tract cancer trial, BILCAP will be submitted as a full proposal to CTAAC for funding in June this year.
A major development from this is the successful bid by the Trials Unit to run multi-centre controlled trials fostered by the British Association for the Study of the Liver (BASL). BASL is a large liver group with approximately 400 members who promote communication between British workers interested in the liver and its disorders. Through collaboration with BASL the Trials Unit aims to establish a large portfolio of liver cancer trials.

© 2004 Cancer Research UK

### P169

### CONSUMER INVOLVEMENT IN THE NATIONAL CANCER RESEARCH INSTITUTE (NCRI) AND THE NATIONAL CANCER RESEARCH NETWORK (NCRN)

T Stevens , R Wilson , M Stead
NCRN, Leeds, United Kingdom

The Consumer Liaison Group (CLG) of the National Cancer Research Institute (NCRI) is made up of patients carers and support group workers who represent consumers on the 17 NCRI cancer site specific Clinical Study Groups (CSGs) and the four cross-cutting CSGs. The role of consumers actively involved in cancer research is rapidly evolving from this starting position with the CLG taking a prominent role in supporting a range of initiatives with the 34 English regional Cancer Research Networks coordinated by the National Cancer Research Network (NCRN). Consumers are also becoming increasingly involved with the work of the Clinical Trials Units, the National Translational Cancer Research Network (NTRAC), and the regional NTRAC Centres. The role of Cancer Voices and Macmillan Cancer Relief in these initiatives has also been significant. Consumer representation in cancer research takes patients and their supporters beyond lobbying or activism into involvement with issues such as ethics, research management, trials design and into evaluating the value of this involvement. The poster maps the various consumer groups in cancer research and describes their relationships with the various regulatory, management and voluntary organisations. Underpinning this work is a belief that studies that consumers have helped design may be studies that patients are more likely to enter. The formation of a new national infrastructure to support research activity and provide a strategic overview of cancer research is providing a unique opportunity to extend and integrate the scope of consumer involvement.

### P170

### GMCCRN 'GREAT EXPECTATIONS' 'GREAT EXPERIENCES'

LE Turner [1], K Cox [2]
[1] Greater Manchester & Cheshire Cancer Research Network, Manchester, United Kingdom, [2] Greater Manchester & Cheshire Cancer Research Network, Manchester, United Kingdom

**Background**: Mortality due to cancer accounts for 25% of men and women in the Northwest. In order that advances in cancer management are achieved more quickly there needs to be an increase in the level of cancer patients being entered into clinical trials. However, it has been reported that only 2-5% of eligible cancer patients participate in such trials.
The Greater Manchester & Cheshire Cancer Research Network (GMCCRN) is the largest network in the country with a population of 3.2 million. Appointment of the research team, both research nurses and clinical trial administrators (CTAs) took place from 7$^{th}$ July to 4$^{th}$ November 2003. Within the networks' 12 NHS trusts there exists a research team to help set up cancer clinical trials, establish an appropriate trial portfolio, support the recruitment and retention of trial patients and act as patient advocate. Anecdotally, recruited patients within GMCCRN have expressed that having a research nurse to manage their care pathway through out a trial has been highly beneficial.
**Aim**: To reach the national accrual proportion of 7.5% of all cancer patients to be recruited into NCRN cancer clinical trials by March 2004. Equating to 900 patients within GMCCRN. To carry out a retrospective case study to examine patient's attitudes and satisfaction in taking part in a clinical trial.
**Results**: At the end of December 2003 the GMCCRN accrual data showed an actual percentage of 6.32%. It is anticipated that the GMCCRN is likely to meet and potentially exceed the 7.5% target.
**Discussion**: Our network clearly demonstrates an impact on the provision of clinical trials for cancer patients. Naturally, the size of the network and the diversity of experienced nurses with or without oncology and research background have played a huge role in the network's success. How patients entering NCRN clinical trials perceive this impact, will be illustrated.

### P171

### FOLLOW UP AFTER COLORECTAL SURGERY (THE FACS TRIAL)

A Corkhill [1], JN Primrose [1], D Mant [2], The FACS study group [1]
[1] University of Southampton, Southampton, United Kingdom,
[2] University of Oxford, Oxford, United Kingdom

The value of follow up after potentially curative colorectal cancer surgery is uncertain although a number of underpowered studies point to the potential value of CEA monitoring and cross sectional imaging. The FACS trial aims to study 5000 patients randomised to one of 4 follow up strategies: Arm 1. Symptomatic follow up in primary care, Arm2. CEA measurement in primary care, Arm 3. CT imaging based hospital follow up, Arm 4. Combination of arms 2 and 3. All detected recurrences are managed by MDTs which include access to liver and lung resectional surgery. The Trial is funded by the NHS R&D Health Technology Assessment Programme.
The trial has completed its pilot phase in 6 hospital sites. Eligibility was lower than anticipated with only 50% of patients being eligible rather than the 84% predicted. This was mainly due to the discovery of metastatic disease when patients were fully staged. Approximately 50% of those eligible approached consented to randomisation. A sample of 31 patients who completed a non participation questionnaire revealed that 4 declined because they did not wish arm 1 (one of these also did not want arm 4), 8 did not want to have hospital follow up (one of these also did not want arm 1) and 13 (42%) were not prepared to participate in any research study. Experience with the pilot also revealed excessive unnecessary delays related to LREC/R&D/Research governance and Primary Care requirements. On one occasion, this resulted in a 2 year delay in setting up a centre.
The FACS trial is feasible and acceptable to patients although there is evidence of a reduced willingness of patients to participate in clinical trials in general. On the basis of the pilot the full study will involve 100 trial sites each aiming to recruit around 2 patients per month. The trail is now open to recruit additional centres.

### P172

### PILOT 'PROOF OF CONCEPT' STUDY OF ELECTRONIC REMOTE DATA CAPTURE (ERDC) APPLIED TO AN ACADEMIC, MULTI-CENTRE, PHASE III ONCOLOGY CLINICAL TRIAL

MCM Jones , RM Harpin
National Cancer Research Network, Leeds, United Kingdom

It has been identified that the entry of patients into clinical trials could be expedited through the use of eRDC technology. A pilot study to start investigating this claim has been carried out to assess the viability of using ICT to support clinical trials and develop an iterative programme for a national rollout. The purpose of the pilot was to demonstrate 'proof of concept' that eRDC could be successfully employed for the collection and storage of real patient information for an academic, multi-centre, Phase III clinical trial. This was achieved from a user perspective by using eRDC to capture parallel data on an existing trial and a technical evaluation of a secure hosted managed service (with benchmarking of software applications to ensure 'value for money'). The potential benefits to be gained from using such technology are improved speed, quality and security of clinical trial information. This is achieved by cutting the time involved in CRF authoring, database development and protocol launch. By using data validation data discrepancies are minimised which in turn reduces time to data locking. Data is available in real time for interim analysis and review. There is a reduction in the need for data entry staff, highly qualified computer programmers, research staff and storage for paper Case Report Forms (CRFs). As these benefits are realised time can be spent more efficiently on accruing patients into trials and potentially, more trials could be run. The use of eRDC was tested alongside standard paper-based systems for the COMICE trial. Complete consideration was given to security, integration with existing systems and IT capability prior to the application being piloted across the networks. This project had a very positive user experience and satisfied the ethical and security requirements of the Caldicott Guardians and DPA. A Phase 2 is planned and will be a multi-centre/multi-trial study to demonstrate scalability and will explore the full functionality of a Clinical Data Management System (CDMS).

© 2004 Cancer Research UK

### P173
### PATIENT ELIGIBILITY SCREENING AND NURSING WORKLOAD IN A TRIALS ACTIVE CANCER CENTRE
S H Shankland , L A Batt , T S Maughan, Velindre Cancer Centre, Cardiff, UK

**Background -** A recent audit in Cardiff highlighted that 16% of new breast cancer patients entered a clinical trial, but this figure is not reproduced in other disease sites. In January 2002 patient screening forms and clinic diaries were introduced at the Velindre Hospital Cancer Centre to record details of all patients potentially eligible for trials and to estimate how long research nurses spent with patients managing the range of clinic visits required.
**Methods -** Data were collected over an eight week period by research nurses.
Screening Forms
Completed for patients in all major disease sites.
Research team screened notes before each clinic.
Highlighted potential trial patients.
Information to clinicians as a prompt to approach patients.
Clinic Diaries
Captured the clinic activity of 9 research nurses.
Categorised visits - informed consent, treatment or follow up.
Time spent per patient was recorded.
**Results** - Commercial studies required approximately 4 times as long for Informed Consent (2hrs 6mins vs 34 mins) as publicly funded studies. On treatment visits for commercial studies take twice as long (35 mins vs 17 mins). No difference was observed in the length of time follow up visits took (18-20 mins). The trials included in the audit ranged from very simple to much more complex.
Conclusions and Further Research
Average times have been calculated but in practice the complexity of each individual trial has to be considered in order to generalise these data.

### P174
### ASSESSING CANCER TRIAL COMPLEXITY AND ITS IMPACT ON WORKLOAD
LA Batt , LK Branston , TS Maughan, Velindre Cancer Centre, Cardiff, UK

**Introduction -** The current portfolio of oncology trials open to recruitment in Wales includes a broad range of trial designs from genetic and epidemiological studies to palliative trials in advanced cancer. When comparing recruitment rates between hospitals, WCTN wanted to account for the levels of complexity of the trials open at each hospital.
Trial complexity has an impact on:
Where a trial can be run - Cancer Unit versus Cancer Centre
Research staff workload
Demands on hospital departments – radiology, pharmacy, nursing
Ethical, R&D and costing requirements
**Methods -** We reviewed 46 trials that had recruited > 5 patients in 2002. A scoring system was devised to reflect the procedures, tests, data collection and design of each protocol. Protocols were used as a reference and details checked with research staff running the trial.
20 parameters were defined and scored according to the workload involved. The maximum possible score was 37.
**Results -** Total complexity scores ranged from 5 - 27. These crude scores were banded in ranges 1 (least complex) – 5 (most complex).
**Conclusions and Further Work -** An assessment of trial complexity is essential when reviewing research units' output and comparing accrual rates. Access to NHS facilities affects the type of trials that can be run and the UK portfolio needs to cater for cancer centres and units. This analysis suggests the most complex trial is approximately 5 times more involved than the simplest study but this may be an underestimate. Our results need review by NCRN networks. Complexity measures would facilitate performance management and workforce planning.

### P175
### A SURVEY OF U.K CANCER CENTRES USE OF TOTAL BODY IRRADIATION
N van As , V Wolstenholme , G Mikhaeel
Guys and St Thomas', London, United Kingdom

Total body irradiation (TBI) is used for a number of different conditions. It can be associated with significant morbidity. There are no clear guidelines as to how TBI should be prescribed or delivered, nor how patients should be followed up. In our survey we look at practice, technique and follow up procedure in UK cancer centres.
We contacted all the centres in the UK by telephone to find out which centres offer TBI. Of the 56 cancer centres in the UK, 31 offer TBI. All centres offering TBI were then sent questionnaires. The questionnaires were divided into two sections. The first section was aimed at the consultants prescribing and responsible for TBI. The second section was aimed at the physicists who are involved in TBI.
The questionnaires were sent in December 2003, and to date we have received 66% of the questionnaires. We intend to follow up by telephone calls and sending second questionnaires to improve the response rate those who have not responded by the end of February.
Interim analysis of the results revealed some interesting findings. All patients are assessed and consented by Clinical Oncology consultants prior to TBI, but despite this, very few Clinical oncologists follow up patients after TBI.
We examined a number of different issues including indications, dose, fractionation, field arrangement, patient position, use of bolus and assessment of dosimetry. To date, the preliminary data is very interesting and we look forward to reporting our full data.

### P176
### THE PIMS TRIAL: A RANDOMISED CONTROLLED TRIAL OF PELVIC IMRT FOR MULTIPLE TUMOUR SITES
J N Staffurth [1], E Hall [3], M Powell [4], V Khoo [2], D Tait [2], M Bidmead[2], D Dearnaley [2], P Wells [4], P Blake [2], M Sydenham [3], PIMS Trial Development Group [1]
[1] Egypt, Velindre Hospital, Cardiff, [2] United Kingdom, Royal Marsden NHS Trust, London, [3] United Kingdom, Institute of Cancer Research, London, [4] United Kingdom, Barts and the London NHS Trust, London

**Introduction:** The incidence of toxicity from whole pelvic radiotherapy (WPRT) is related to the volume of bowel irradiated to high doses. Preclinical studies of IMRT for WPRT have shown a reduction in the volume of bowel irradiated to high dose, which may lead to reduced toxicity. Initial clinical experience from phase 1 trials and non-randomised series has been encouraging. We are designing a randomised trial to investigate the ability of IMRT to reduce the toxicity of WPRT for prostate, gynaecological and rectal tumours.
**Study design:** Phase III multi-centre, non-blinded randomised trial, stratification by tumour site and centre. Planned sample size of 600 patients randomised in a 1:1 ratio to the IMRT or conformal-RT arm.
**Endpoint:** Primary endpoint is late gastrointestinal radiation toxicity: Grade 2 or greater using NCI CTC v. 3.0 Late Radiation Morbidity Score. Secondary endpoints are late genitourinary radiation toxicity, acute radiation toxicity, quality of life, tumour control and survival.
**Entry criteria:** Locally advanced **Prostate** adenocarcinoma with ≥30% estimated risk of pelvic nodal involvement. **Cervical** carcinoma with high risk of pelvic failure after TAH (+/-lymphadenectomy). **Endometrial** adenocarcinoma with high risk of pelvic failure after TAH (+/-lymphadenectomy). **Rectal** adenocarcinoma suitable for long-course pre-operative (chemo-) radiation.
**Treatment protocol:** Target volumes and organs at risk to be outlined on every CT slice in both arms; patients in the control arm will be treated with conformal radiotherapy. The same biologically effective doses will be used in both arms of the study. Standard neoadjuvant, concurrent and adjuvant therapies will be the same for each arm (defined by tumour subgroup).
**Quality Assurance:** An emphasis will be made to develop a quality assurance program to ensure accuracy of outlining, planning and delivery throughout the trial.

© 2004 Cancer Research UK

### P176:1

### THE MARS (MESOTHELIOMA AND RADICAL SURGERY) TRIAL

MA Sydenham [1], C Peckitt [1], S Piggott [1], J Peto [1], D Waller [3], T Treasure [2], MARS Trial Management Group [1]
[1] United Kingdom, Institute of Cancer Research, Sutton, [2] United Kingdom, Guys and St Thomas' Hospital, London, [3] United Kingdom, Glenfield Hospital, Leicester

**Introduction:** The role of radical surgery in mesothelioma has never been tested in a randomised trial. Cancer Research UK has given funding for a 50 patient pilot study.
**Pilot Study:** The pilot study will determine the feasibility and acceptability of performing a randomised trial comparing extra-pleural pneumonectomy (EPP) against no EPP surgery within the context of trimodality therapy (chemotherapy, surgery and post-operative radiotherapy).
**Methodology for Pilot Study:** Agreement of the study design has been difficult and contentious. Consensus has been reached and the protocol submitted for MREC approval. Patients with potentially operable disease on staging will first be registered at the local centre, where they will have 3 cycles of cisplatin based chemotherapy. The patients will then be restaged following the chemotherapy. The local centre, surgical centre and MARS Multi Disciplinary Teams will assess the final eligibility. Patients eligible for surgery will be randomised to EPP or no EPP surgery. Patients randomised to receive EPP will be referred to a designated surgical centre. After surgery patients will be given radical hemithoracic radiotherapy. Patients randomised to no EPP surgery will receive active oncological management at the local centre. Quality of life will be measured at various time points throughout the trial.
**Main Trial:** If the pilot study is successful it will lead to a main trial, the aim of which is to determine if surgery is beneficial, in terms of survival & quality of life. The target is to recruit 700 patients over 3 years into a Europe-wide trial.

### P176:2

### PROSPECTIVE RANDOMISED TRIAL TESTING 5.7GY AND 6.0GY FRACTIONS OF WHOLE BREAST RADIOTHERAPY IN WOMEN WITH EARLY BREAST CANCER ('FAST' TRIAL)

D Bloomfield [4], M Sydenham [1], J LeVay [3], I Syndikus [5], M Hatton [6], P Bliss [7], P Dyson [8], S Kelly [9], R Agrawal [10], P Barrett-Lee [11], C Abson [12], JR Owen [13], S Kumar [14], M Brunt [15], L Whipp [16], M Illsley [17], J Bliss [1], J Yarnold [2]
[1] United Kingdom, Institute of Cancer Research, Sutton, [2] United Kingdom, Royal Marsden Hospital, Sutton, [3] United Kingdom, Ipswich Hospital, Ipswich, [4] United Kingdom, Royal Sussex County Hospital, Brighton, [5] United Kingdom, Clatterbridge Centre for Oncology, Bebbington, [6] United Kingdom, Weston Park Hospital, Sheffield, [7] United Kingdom, Torbay Hospital, Torbay, [8] United Kingdom, Cumberland Infirmary, Carlisle, [9] United Kingdom, Derriford Hospital, Plymouth, [10] United Kingdom, Royal Shrewsbury Hospital, Shrewsbury, [11] United Kingdom, Velindre Hospital, Cardiff, [12] United Kingdom, Kent Oncology Centre, Maidstone, [13] United Kingdom, Gloucestershire Oncology Centre, Cheltenham, [14] United Kingdom, Cookridge Hospital, Leeds, [15] United Kingdom, Stafford General Hospital, Stoke on Trent, [16] United Kingdom, Bristol Haematology and Oncology Centre, Bristol, [17] United Kingdom, Royal Surrey County Hospital, Guildford

**Background:** There is a resurgence of interest in the potential biological and clinical benefits of fewer, larger radiotherapy fractions for breast radiotherapy.
**Aim:** To test 5 fractions of 5.7Gy and 6.0Gy against 25 fractions of 2.0Gy in terms of late normal tissue effects and tumour control in women prescribed whole breast radiotherapy (no boost) after local excision of early breast cancer in a prospective randomised controlled clinical trial.
Inclusion criteria: Age ≥50 years, invasive carcinoma breast, breast conservation surgery, pathological tumour size <3.0 cm, complete microscopic resection, axillary node negative
Exclusion criteria: Mastectomy, lymphatic radiotherapy, radiotherapy breast boost, neoadjuvant or adjuvant cytotoxic therapy.
Randomisation:

| | |
|---|---|
| Control arm: | 50.0Gy in 25 fractions of 2.0Gy in 35 days |
| Test arm 1: | 30.0Gy in 5 fractions of 6.0Gy in 35 days |
| Test arm 2: | 28.5Gy in 5 fractions of 5.7Gy in 35 days |
| Test arm 3: | 30.0Gy in 5 fractions of 6.0Gy in 15 days |
| Test arm 4: | 28.5Gy in 5 fractions of 5.7Gy in 15 days |

**Radiotherapy delivery:** Tangential fields to the whole breast; no boost; with full dose compensation
**Primary endpoint:** Change in photographic breast appearance.
**Secondary endpoint:** Tumour recurrence in the breast.
Sample size: 1500
**Discussion:** The aim is to complete accrual into this pilot study by September 2005 (before the IMPORT trial starts). A proposal based on 2-year outcome data will be submitted in 2006 for a phase 3 trial using tumour control as the primary endpoint. The goal is to evaluate a 5-fraction schedule of intensity modulated radiotherapy as the standard of care for this large population of patients by 2010.

### P176:3

### RANDOMISED TRIAL TESTING INTENSITY MODULATED RADIOTHERAPY AND PARTIAL ORGAN RADIOTHERAPY IN EARLY BREAST CANCER (IMPORT TRIAL)

C Coles [3], E Donovan [2], K Venables [4], C Rawlings [5], H Mayles [6], S Bentzen [7], M Sydenham [1], J Bliss [1], J Yarnold [2], IMPORT Working Party [1]
[1] United Kingdom, Institute of Cancer Research, Sutton, [2] United Kingdom, Royal Marsden Hospital, Sutton, [3] United Kingdom, Addenbrookes Hospital, Cambridge, [4] United Kingdom, Mount Vernon Hospital, Northwood, [5] United Kingdom, Torbay Hospital, Torbay, [6] United Kingdom, Clatterbridge Centre for Oncology, Bebbington, [7] United Kingdom, Gray Cancer Institute, Northwood

**Background:** Standard radiotherapy aims to distribute dose evenly across the breast, even though local tumour recurrence is concentrated in the index quadrant. Dose intensification is achieved by giving additional fractions (sequential boost). Dose intensity modulation adjusted to individual recurrence risk and to the pattern of relapse in the breast would be much more effectively achieved by dose intensity modulation across the breast and fewer fractions.
**Aim:** To test dose intensity modulation and partial breast radiotherapy after conservation surgery for early stage breast cancer.
**Eligibility:** Age 18 years and above, operable unilateral breast cancer (T1-3, N0-1, M0) at presentation, breast conserving surgery, histological confirmation of invasive carcinoma, complete microscopic resection, written informed consent and availability for follow-up.
Study design: The control arm delivers 40Gy/15 fractions (F) to whole breast volume; Sequential boost 16Gy/8F to tumour bed, if indicated. The test arm delivers: 36Gy/15F to low dose breast volume (outside index quadrant); 40Gy/15F to standard dose breast volume (includes index quadrant); Concomitant boost 48Gy/15F or 53Gy/15F to tumour bed, if indicated.
**Primary endpoint:** Local tumour control. Secondary endpoints include location of tumour relapse in breast, metastases, late adverse effects in normal tissues, quality of life and economic evaluation.
**Sample size:** 4000 patients will provide 90% power to detect a difference of 3% in 5-year recurrence rates (assuming a 10% recurrence rate at 5 years in the control arm, 5% rate of loss by 5 years follow-up).
**Discussion:** Modulation of fraction size promises to be superior to adjustment of fraction number for risk-adapted radiotherapy. The results of the proposed IMPORT trial combined with the results of the START and FAST trials are expected to underpin future 3D intensity modulated breast radiotherapy delivered in 5 fractions over 5 – 15 days as the standard of care for up to 20,0000 women per year in the UK.

### P176:4

### THE PROTECT TRIAL - EVALUATING THE EFFECTIVENESS OF TREATMENT FOR CLINICALLY LOCALISED PROSTATE CANCER (ISRCTN20141297)

JA Lane [1], FC Hamdy [2], DE Neal [3], JL Donovan [1], The ProtecT study group [1]
[1] United Kingdom, University of Bristol, Bristol, [2] United Kingdom, University of Sheffield, Sheffield, [3] United Kingdom, University of Cambridge, Cambridge

**Aims and Methods:** The ProtecT study is a UK multi-centre randomised controlled trial of the effectiveness, cost-effectiveness and acceptability of treatments for clinically localised prostate cancer preceded by case finding in general practices. The trial compares three treatments: active monitoring by PSA (prostate specific antigen), radical prostatectomy and radical radiotherapy. Qualitative research is used extensively to investigate methods of giving information to participants as well as in-depth case studies of participants' experiences[3]. The primary outcome is survival at 10 years with secondary outcomes of disease progression, treatment complications, urinary symptoms, sexual function, QoL and health service utilisation.
Results: The study commenced in 1999 and will recruit over 100,000 unselected men. There are nine centres recruiting across the UK. Currently, over 33,000 men have been recruited (50% of those invited) and 2,884 have a raised PSA test (10.0%). There are 823 men with prostate cancer (25% of those with a raised PSA) of which 622 have clinically localised disease and 460 men have been randomised
Extensive qualitative research has increased randomisation rates from less than 50% at the start of the study to over 70% currently. Analyses of the qualitative data has helped improve information given to participants as well as patient information sheets.
**Conclusions:** This large randomised trial focuses on an area of clinical uncertainty around the most appropriate treatment for localised prostate cancer. Major insights into the conduct of randomised trials have been gained through the novel use of qualitative research methods. The results of this trial will inform UK policy for prostate cancer treatment.
References
1. Majeed A, Babb P, Jones J, Quinn M. *BJU Int* 2000; 85:1058.
2. Selley S, Donovan JL,et al. *HTA. Programme: HTA*, 1997; 1 (2).
3. Donovan JL, Mills N, Smith M, et. al. *BMJ*. 2002; 325: 766.

© 2004 Cancer Research UK

